# Identification, evolution and expression analyses of whole genome-wide *TLP* gene family in *Brassica napus*

**DOI:** 10.1186/s12864-020-6678-x

**Published:** 2020-03-30

**Authors:** Tong Wang, Jingjing Hu, Xiao Ma, Chunjin Li, Qihang Yang, Shuyan Feng, Miaomiao Li, Nan Li, Xiaoming Song

**Affiliations:** 1 College of Life Sciences, North China University of Science and Technology, 21 Bohai Road, Caofeidian Xincheng, Tangshan, 063210 Hebei China; 2 Library, North China University of Science and Technology, Tangshan, 063210 Hebei China

**Keywords:** *TLP* gene family, Polyploid, Orthologous and paralogous, Gene duplication and loss, Expression analysis, *B. napus*

## Abstract

**Background:**

*Brassica* is a very important genus of Brassicaceae, including many important oils, vegetables, forage crops, and ornamental horticultural plants. *TLP* family genes play important regulatory roles in the growth and development of plants. Therefore, this study used a bioinformatics approach to conduct the systematic comparative genomics analysis of *TLP* gene family in *B. napus* and other three important Brassicaceae crops.

**Results:**

Here, we identified a total of 29 *TLP* genes from *B. napus* genome, and they distributed on 16 chromosomes of *B. napus.* The evolutionary relationship showed that these genes could be divided into six groups from Group A to F. We found that the gene corresponding to *Arabidopsis thaliana AT1G43640* was completely lost in *B. rapa*, *B. oleracea* and *B. napus* after whole genome triplication. The gene corresponding to *AT1G25280* was retained in all the three species we analysed, belonging to 1:3:6 ratios. Our analyses suggested that there was a selective loss of some genes that might be redundant after genome duplication. This study proposed that the *TLP* genes in *B. napus* did not directly expansion compared with its diploid parents *B. rapa*, and *B. oleracea*. Instead, an indirect expansion of *TLP* gene family occurred in its two diploid parents. In addition, the study further utilized RNA-seq to detect the expression pattern of *TLP* genes between different tissues and two subgenomes.

**Conclusions:**

This study systematically conducted the comparative analyses of *TLP* gene family in *B. napus*, discussed the loss and expansion of genes after genome duplication. It provided rich gene resources for exploring the molecular mechanism of *TLP* gene family. Meanwhile, it provided guidance and reference for the research of other gene families in *B. napus*.

## Background

*B. napus* belonged to the *Brassica* genus, which included many important oils, vegetables crops and ornamental horticultural plants. The allotetraploids *B. napus* (*Brassica napus*; AACC, 2n = 38) was obtained by crossing of the two diploid basic species of *B. rapa* (*Brassica rapa*; AA, 2n = 20), and *B. oleracea* (*Brassica oleracea*; CC, 2n = 18) [1–3]*. B. napus* was not only one of the world’s four major oil crops, but also one of the most important oil crops in China. Currently, the genomes of these species have been sequenced and the datasets have been released [2, 4–6]. Recently, several important achievements and progress in comparative genomics and functional genomics research have been achieved, which reflected the importance and practicality of these data [7–9]. Therefore, we could use bioinformatics to dig deeper into these public data. Until now, the *TLP* gene family of *B. napus* has not been reported at the genome level.

The Tubby-like proteins (*TLP*) family was a smaller gene family in animals, it played very important role in animal growth and development [10, 11]. The *Tubby* gene was first isolated by positional cloning in obese mice, subsequently, other members of *TLP* gene family were successively identified [10, 12]. Studies have shown that following activation of G protein subsets by phospholipase C-β, mouse *Tubby* was transferred from the cytoplasmic membrane to the center [13, 14]. *TLP* gene family members contained a tubby domain about 270 amino acids in the C-terminal, and a plurality of different domains in the N-terminal. Diversity of the N-terminal indicated the diversity functions of *TLP* genes [11, 15]. In 1999, Shapiro Lab published the crystal structure of the tubby domain, laying the foundation for studying its function [16].

The spatial structure of the tubby domain consisted of a hydrophobic α-helix and a 12-fold inverted β-fold. The hydrophobic α-helix was located at the C-terminus of TLP protein [16, 17]. Unlike the diversity of N-terminal structures in animals, the N-terminus of TLP protein in plants often contained a conserved F-box domain [16, 18]. This F-box domain was first described as a sequence motif of cyclin F, and it interacted with the protein S-phase kinase-associated protein 1 (SKP1). Experimental results indicated that SKP1 could bridge different F-box proteins to CDC53(Cullin), forming the designated SKP1/Cullin/F-box (SCF) complexes, which function in recognizing of target proteins specifically for ubiquitin-dependent proteolysis. F-box proteins regulated different biological processes, including cell cycle cycling, translational control, and signal transduction. For example, *TIR1* was involved in auxin response during plant growth and development, and *UFO* was critical in flower organ identity determination [19–21], *COI1* participated in jasmonic acid mediated defense response [22, 23], and *ZTL* or *FKF1* control circadian clock [24, 25].

The *TLP* genes were widespread in many plants [26]. In *Oryza sativa*, *A. thaliana*, *Zea mays*, *Malus domestica*, *Cicer arietinum* and other plants, a genome-wide *TLP* gene family has been studied [27–30]. However, it has not been reported in *Brassica* crops, especially in *B. napus*. Therefore, this study used bioinformatics tools to conduct the comprehensively analyses of *Brassica TLP* gene family, including identification, gene structure, chromosomal distribution, orthologous and paralogous, duplication and loss, and expression pattern analyses at the genome level. Furthermore, comparative analyses were conducted with its two native parents (*B. rapa* and *B. oleracea*) and *A. thaliana*. This study will lay the foundation for further investigating the biological function of this family members in *B. napus*. At the same time, it provided a methodological reference for studying this gene family in other oil crops and related species.

## Results

### Identification and comparative analysis of *TLP* gene family in *B. napus*

**Fig. 1:**
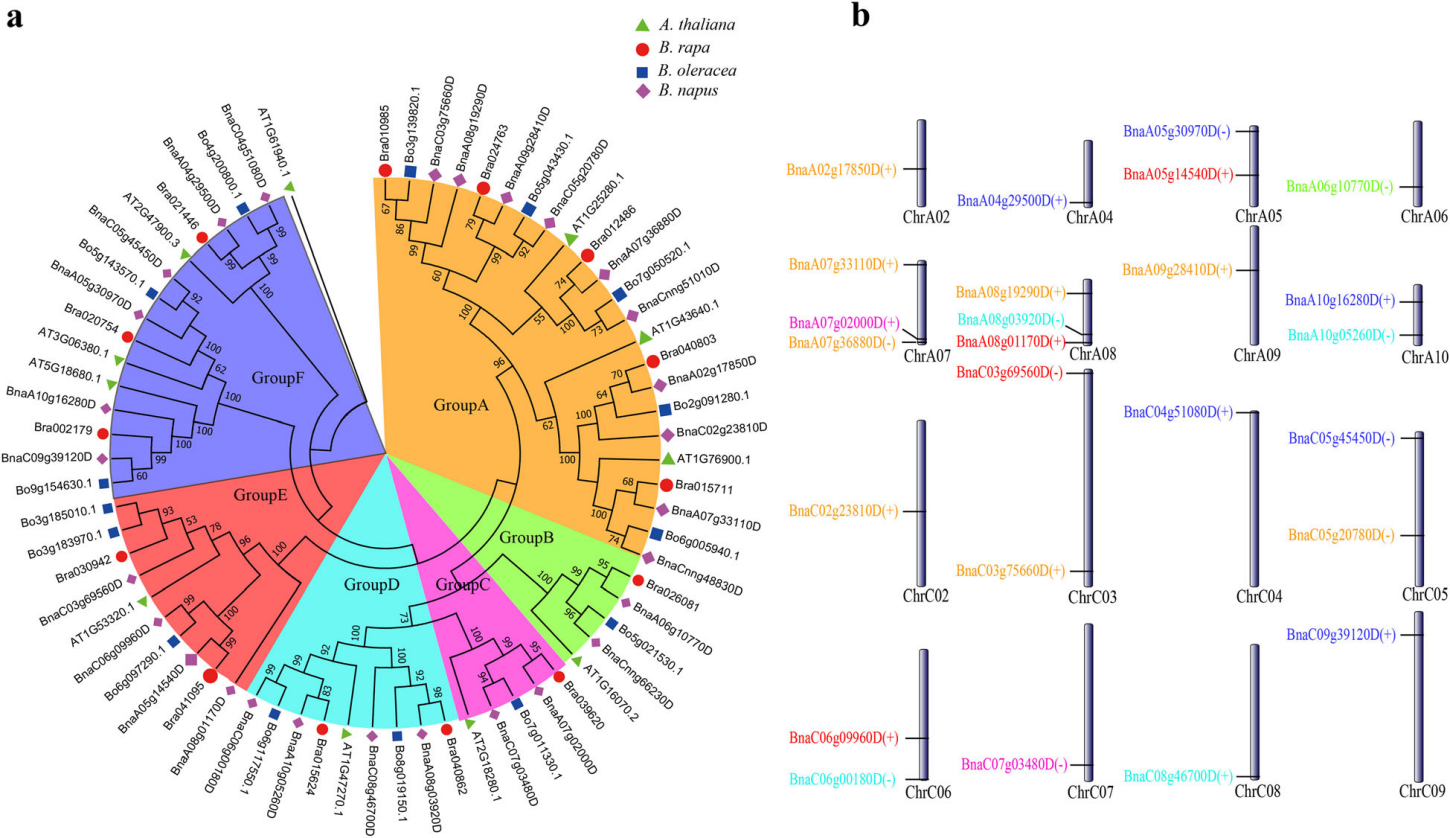
Phylogenetic relationship and chromosome distribution analyses of *TLP* gene family. **a** The construction of phylogenetic tress using the *TLP* gene family among *B. napus, B. rapa, B. oleracea, and A. thaliana.* Phylogenetic tree topology was generated by MEGA7.0. For the major nodes, neighbour-joining (NJ) bootstrap values above 50% are shown. The Groups A to F indicate the groups obtained by bootstrap values and phylogenetic topology. **b** The distribution of *B. napus TLP* transcription factors on chromosomes. The genes with different colors correspond to above mentioned 6 groups on phylogenetic tree

Totally, 29 *TLP* transcription factor members were identified from the whole genome of *B. napus* using bioinformatics methods (Table 1). Further analysis showed that the domain of gene (*BnaC09g39130D*) was incomplete and removed in the subsequent analysis. In order to explore the structure and biological function of *TLP* family genes in *B. napus*, we compared them with the model plant *A. thaliana.* The results showed that *TLP* family genes of *B. napus* had a high homology with *A. thaliana* corresponding genes (E-value<7E-136 ~ 0), which provided a good guidance for studying the function of *TLP* family genes in *B. napus.* Among the 28 *B. napus* genes identified, *BnaA10g05260D* was the longest, over 4145 bp; *BnaC04g51080D* was the shortest, only 1586 bp (Table 1). To investigate the evolutionary relationship of this family in Brassicaceae crops, we identified 14, 15 and 11 *TLP* family genes from *B. rapa, B. oleracea*. and *A. thaliana,* respectively. The phylogenetic tree was constructed using *TLP* family genes of these four species (Fig. 1a). According to the topology of phylogenetic tree, 28 *BnTLPs* were divided into 6 groups, named Group A to F. It could be seen from the phylogenetic tree that Group A contains the most *TLP* family genes, with 10 genes in *B. napus*, followed by Group F (6), Group D (4), and Group E (4). In Group A, there were 5 genes from subgenome A, and 3 genes from subgenome C.

**Table 1:** The summary of *TLP* gene family members in *B. napus* and compared with *A. thaliana*

*B. napus*	Gene start	Gene end	Gene length	Group	A. *thaliana*	Identity (%)	E-value	Score
*BnaC03g75660D*	4,377,802	4,380,799	2997	A	*AT1G25280.1*	84.56	0	711
*BnaA04g29500D*	1,400,068	1,402,078	2010	D	*AT2G47900.3*	88.29	0	692
*BnaA07g36880D*	741,264	743,428	2164	A	*AT1G25280.1*	88.42	0	730
*BnaA05g30970D*	21,446,109	21,448,431	2322	D	*AT3G06380.1*	76.04	0	587
*BnaC07g03480D*	4,680,016	4,682,051	2035	F	*AT2G18280.1*	86.04	0	629
*BnaC03g69560D*	59,391,825	59,394,400	2575	E	*AT1G53320.1*	91.32	0	622
*BnaA05g14540D*	8,994,374	8,996,696	2322	C	*AT1G53320.1*	86.43	0	619
*BnaC04g51080D*	48,418,230	48,419,816	1586	D	*AT2G47900.3*	87.32	0	654
*BnaC08g46700D*	945,065	946,978	1913	F	*AT1G47270.1*	77.27	0	572
*BnaCnng48830D*	48,277,183	48,280,749	3566	A	*AT1G76900.1*	81.68	0	731
*BnaCnng51010D*	50,479,652	50,481,521	1869	A	*AT1G25280.1*	85.52	0	723
*BnaA06g10770D*	5,654,536	5,656,427	1891	B	*AT1G16070.2*	86.72	0	707
*BnaA08g19290D*	14,882,996	14,886,001	3005	A	*AT1G25280.1*	85.27	0	709
*BnaA09g28410D*	21,293,066	21,295,808	2742	A	*AT1G25280.1*	85.97	0	702
*BnaC02g23810D*	20,887,558	20,890,224	2666	A	*AT1G76900.1*	83.41	0	744
*BnaC09g39120D*	41,813,712	41,815,558	1846	D	*AT5G18680.1*	86.55	7.00E-136	387
*BnaC06g09960D*	11,871,153	11,873,618	2465	C	*AT1G53320.1*	86.7	0	638
*BnaA02g17850D*	10,791,533	10,794,166	2633	A	*AT1G76900.1*	84.13	0	738
*BnaA07g02000D*	1,660,832	1,662,975	2143	F	*AT2G18280.1*	85.53	0	608
*BnaCnng66230D*	65,942,721	65,944,602	1881	E	*AT1G16070.2*	86.22	0	710
*BnaA08g03920D*	3,231,533	3,233,325	1792	F	*AT1G47270.1*	81.27	0	624
*BnaC06g00180D*	279,021	281,085	2064	F	*AT1G47270.1*	84.14	0	651
*BnaA08g01170D*	891,151	893,938	2787	E	*AT1G53320.1*	87.99	0	595
*BnaC05g45450D*	41,346,349	41,348,694	2345	D	*AT3G06380.1*	78.39	0	603
*BnaA10g05260D*	3,001,258	3,005,403	4145	F	*AT1G47270.1*	79.9	0	615
*BnaC05g20780D*	14,250,016	14,252,879	2863	A	*AT1G25280.1*	83.93	0	664
*BnaA07g33110D*	22,783,241	22,786,597	3356	A	*AT1G76900.1*	81.72	0	731
*BnaA10g16280D*	12,422,564	12,424,770	2206	D	*AT5G18680.1*	83.59	0	611

### Chromosome distribution analysis of *TLP* family genes in *B. napus*

To more intuitively understand the distribution of *TLP* family genes on the chromosomes of *B. napus,* we performed a chromosomal localization analysis (Fig. 1b). Since the genomic data of *B. napus* has not yet been fully mapped to the chromosome, the chromosomal location of some genes are still unclear, so these genes are not shown on the map (two genes, *BnaCnng51010D* and *BnaCnng66230D*). The localization information showed that members of this family were distributed in 16 of 19 chromosomes of *B. napus.* There was no *TLP* gene distribution on the three chromosomes of ChrA01, ChrA03 and ChrC01. ChrA07 and ChrA08 chromosomes had the most genes (3 genes). For the same group of genes, they were also distributed on multiple chromosomes, and there was no obvious phenomenon that the genes in the same group were clustered in a certain interval. For example, the six genes in Group F were distributed on six chromosomes. However, the distribution of genes on chromosomes was not uniform. Most genes were distributed at both ends of the chromosome (such as ChrA04, ChrA07, ChrA08, ChrC04, ChrC05, ChrC07, ChrC08), and there were fewer *TLP* genes near the centromere. This may be due to the fact that there are more repeat sequences in centromere, resulting in a small distribution of genes on the whole [31, 32].

### Conservative motif and gene structure analyses of *TLP* gene family

**Fig. 2:**
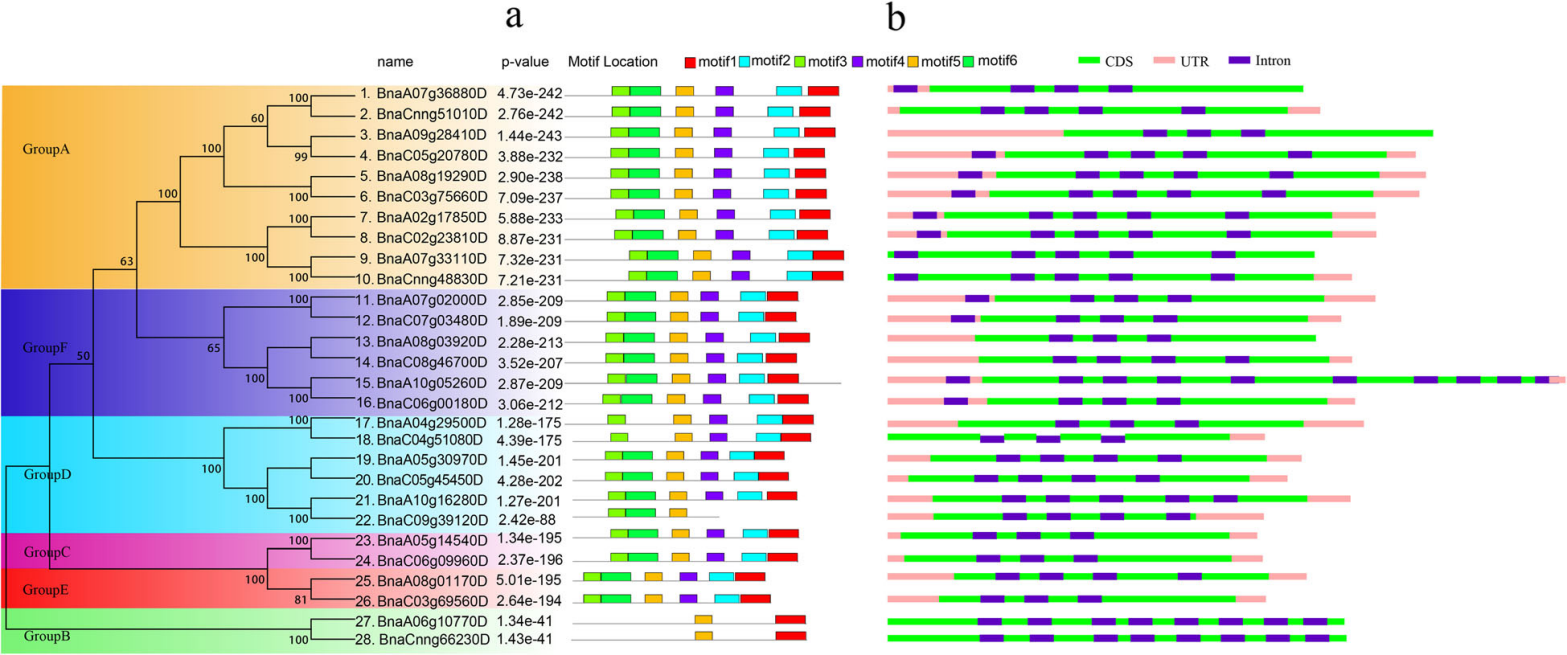
The converted motif and gene structure analyses of *TLP* gene family in *B. napus.* **a** The motif identification of *TLP* gene family in *B. napus*. **b** The gene structure of *TLP* gene family in *B. napus*

The sequence characteristics of 28 *TLP* genes in *B. napus* were analyzed using MEME software (Fig. 2a), and a total of 6 conserved motifs were obtained. The position of motif3 was in the front, and the position of motif1 and motif2 were backward. Twenty-three genes contained all six conserved motifs from motif1 to motif6. *BnaA04g29500D* and *BnaA05g51080D* (GroupD) lacked motif6; *BnaA09g39120D* (GroupD) lacked motif1, motif2 and motif4; *BnaA06g10770D* and *BnaCnng66230D* (GroupB) lacked motif2, motif3, motif4, motif6. The results showed that there was no loss of any conservative motifs in the four groups (GroupA, GroupC, GroupE, and GroupF). Of the 6 genes in GroupD, 3 of them lost part of the conserved motif. We found that motif5 was present in all 28 *TLP* genes in *B. napus,* indicating its presence or absence as a marker for the identification of *TLP* genes. In addition, motif1 was lost only in one gene (*BnaA09g39120D*), and motif3 was lost only in two genes (*BnaA06g10770D* and *BnaCnng66230D*). This indicated that these conserved motifs were relatively conservative and might play a very important role in the function of *TLP* gene family. Taken together, these results indicated that the gene conservation motifs within the group were relatively consistent and had a more consistent positional distribution across the genes.

In the study of molecular evolution, the distribution of introns provided important evidence for the phylogenetic relationship among members of the gene family. Gene structure analysis showed that *TLP* gene family structure of *B. napus* was relatively complex, and each gene contained introns (Fig. 2b). *BnaA06g05260D* contained the most introns and had 10 introns, followed by *BnaA06g10770D* and *BnaCnng66230D* with 8 introns. From the perspective of gene length, *BnaA10g05260D* was significantly longer than other genes. The three genes *BnaA06g10770D*, *BnaCnng66230D* and *BnaA07g33110D* lacked UTR region at two ends, while some genes lacked UTR region at one end. Through gene structure analysis, it was found that the genes in the same group had similar intron/exon distribution patterns. For example, two genes in the GroupB had almost the same genetic structure distribution characteristics.

### Analysis of orthologous and paralogous *TLP* family genes in Brassicaceae crops

**Fig. 3:**
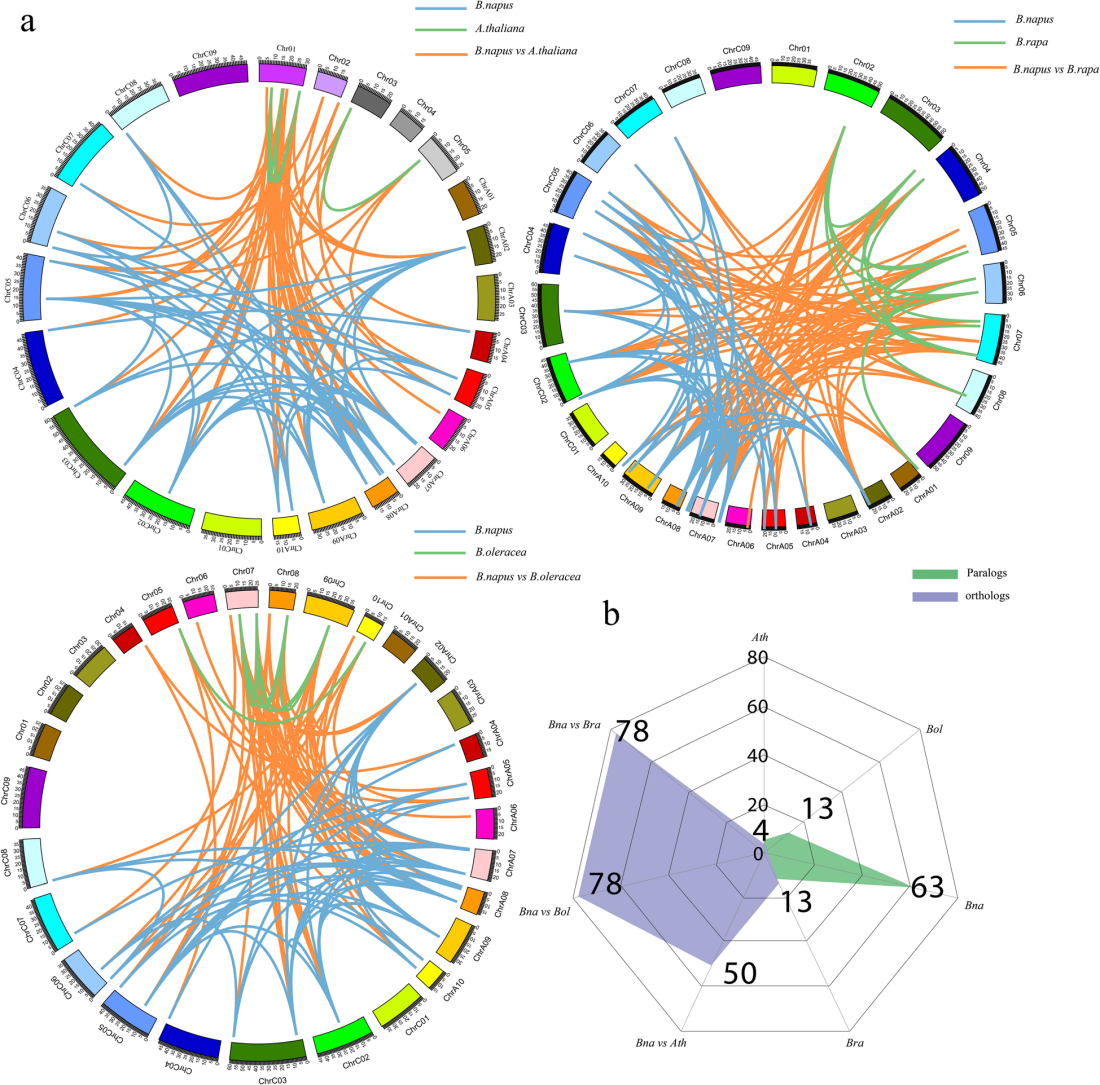
The paralogous and orthologous analyses of *TLP* gene family. **a** The plot of paralogous and orthologous *TLP* gene pairs between *B. napus* and *A. thaliana*, *B. rapa*, *B. oleracea,* respectively. **b** The statistics analysis of paralogous and orthologous of *TLP* gene family among four species

We further analyzed the orthologous and paralogous of *TLP* gene family between *B. napus* and *A. thaliana*, *B. rapa,* or *B. oleracea.* The orthologous and paralogous network maps between *B. napus* and these three species were constructed by Circos program (Fig. 3a). Orthologs referred to genes that have evolved from vertical pedigrees from different species and typically retained the same function as the original gene. Here, 50 pairs of orthologous genes were identified in *B. napus* and *A. thaliana*; 78 pairs of orthologous genes were identified in *B. napus* and two diploid parents, *B. rapa*, *B. oleracea* (Fig. 3b, Table S1). Paralogs referred to genes that were found in the same species and derived from gene duplication, and might evolve new and previously related functions. A total of 4, 13, 13 and 63 pairs of paralogous genes were identified in *A. thaliana, B. rapa*, *B. oleracea* and *B. napus* (Fig. 3b, Table S2).

In addition, the divergence time and selection types of orthologous *TLP* gene pairs were calculated according to the nonsynonymous substitutions (Ka) and synonymous (Ks). To avoid the misalignment, we only used the orthologous gene pairs with Ks < 1 according to previous report [33]. Finally, we obtained the Ks, Ka, Ka/Ks, selection types, and divergence time of 133 orthologous gene pairs (Table S3). The results showed that most of orthologous gene pairs (132/133) had Ka/Ks ratios < 1, indicating purifying selection on these orthologous *TLP* gene pairs. Furthermore, we estimated the divergence time of orthologous *TLP* gene pairs according to synonymous substitution rate (Table S3). The results indicated that the divergence time was 12.81~31.89 million years ago (Mya) for 28 orthologous *TLP* gene pairs between *B. napus* and *A. thaliana*. Based on the divergence time (14.5 Mya) of *B. napus* and *A. thaliana*, 22 and 6 orthologous genes pairs were formed before and after the divergence of *B. napus* and *A. thaliana*, respectively. The divergence time was from 0.12 to 29.80 Mya for the orthologous *TLP* gene pairs between *B. napus* and *B. oleracea*. Based on the divergence time (0.045 Mya) of *B. napus* and *B. oleracea*, all 52 orthologous genes pairs were formed before the divergence of *B. napus* and *B. oleracea*. Similar, the divergence time of orthologous *TLP* gene pairs was 0.25~32.14 Mya between *B. napus* and *B. rapa*. Based on the divergence time (0.045 Mya) of *B. napus* and *B. rapa*, all 53 orthologous genes pairs were formed before the divergence of *B. napus* and *B. rapa.*

### Duplicated type identification and synteny analyses of *B. napus* and other 3 species

The gene duplications have contributed to the expansion of gene family. We examined 5 types of gene duplications: singleton, dispersed, proximal, tandem, and WGD or segmental duplication by MCScanX program (Table 2, Table S4). Here, we found evidence that WGD likely contributed most to the expansion of this gene family in *B. napus* and *B. oleracea.* The percentage of WGD was 82.1% in *B. napus, B. rapa* (35.7.0%), *B. oleracea* (80.0%), and *A. thaliana* (18.2%) (Table 2). However, dispersed duplication contributed most to gene expansion in *B. rapa* (64.3%) and *A. thaliana* (72.7%). No proximal and tandem duplication were detected for *TLP* gene family among these four species. Actually, by checking gene collinearity within a genome, we found that 82.1, 35.7, 80.0 and 18.2% of *TLP* genes were located in collinear blocks for *B. napus*, *B. rapa*, *B. oleracea*, and *A. thaliana,* respectively (Table 3). The percentage of *TLP* genes located in the collinear blocks was significantly larger than the average genome-wide level for *B. napus* and *B. oleracea.*

**Table 2:** The identification of duplicated type for *TLP* genes and all genes in *B. napus* and other three Brassicaceae species

Species	Singleton		Dispersed		Proximal		Tandem		WGD or segmental			Total	
	Genome	*TLP*	Genome	*TLP*	Genome	*TLP*	Genome	*TLP*	Genome	*TLP*	Percentage	Genome	*TLP*
*B. napus*	7768	0	26,907	5	2428	0	2708	0	61,229	23	82.1%	101,040	28
*B. rapa*	3666	0	10,622	9	873	0	2369	0	23,489	5	35.7%	41,019	14
*B. oleracea*	4807	0	25,232	3	2515	0	2523	0	24,148	12	80.0%	59,225	15
*A. thaliana*	5156	1	10,670	8	1046	0	3026	0	7519	2	18.2%	27,417	11

**Table 3:** The synteny analyses of *TLP* genes and all genes in *B. napus* and other three Brassicaceae species

Species	All genes				*TLP genes*			
	Total collinear blocks	Gene number in collinear blocks	Total genes	Percentage (%)	Collinear blocks contained *TLP* gene	*TLP* gene in collinear block	Total *TLP* genes	Percentage (%)
*B. napus*	2914	61,229	101,040	60.6	24	23	28	82.1
*B. rapa*	650	23,489	41,019	57.3	4	5	14	35.7
*B. oleracea*	747	24,148	59,225	40.8	8	12	15	80.0
*A. thaliana*	216	7519	27,417	27.4	1	2	11	18.2

### Expansion analysis of *TLP* gene family in *Brassica* species

In order to further explore whether the expansion of *TLP* gene family in *B. napus* was a direct or indirect expansion, we conducted a more detailed analysis. In general, for most genome-wide replication events, including WGD (whole genome duplication) and WGT (whole genome triplication), replication was accompanied by loss of genes [34, 35]. To elucidate the evolution of *TLP* gene family in *Brassica*, we performed gene loss and replication retention analysis. Compared with *A. thaliana*, a WGT and hybridization event occurred in *B. napus* after differentiation with *A. thaliana* [4–6]. Here, 11 *TLP* family genes were identified in *A. thaliana*. In theory, there should be 66 TLP genes in *B. napus* (11 × 3 × 2), while only 28 *TLP* genes were identified in *B. napus*. Although a WGT event occurred after the differentiation of *Brassica* species and *A. thaliana*, the number of *TLP* genes did not increase significantly. There were only 14 and 15 genes in *B. rapa* and *B. oleracea* species, indicating that this WGT event did not result in a significant expansion of the *TLP* gene, or a gene loss occurred after expansion.

**Fig. 4:**
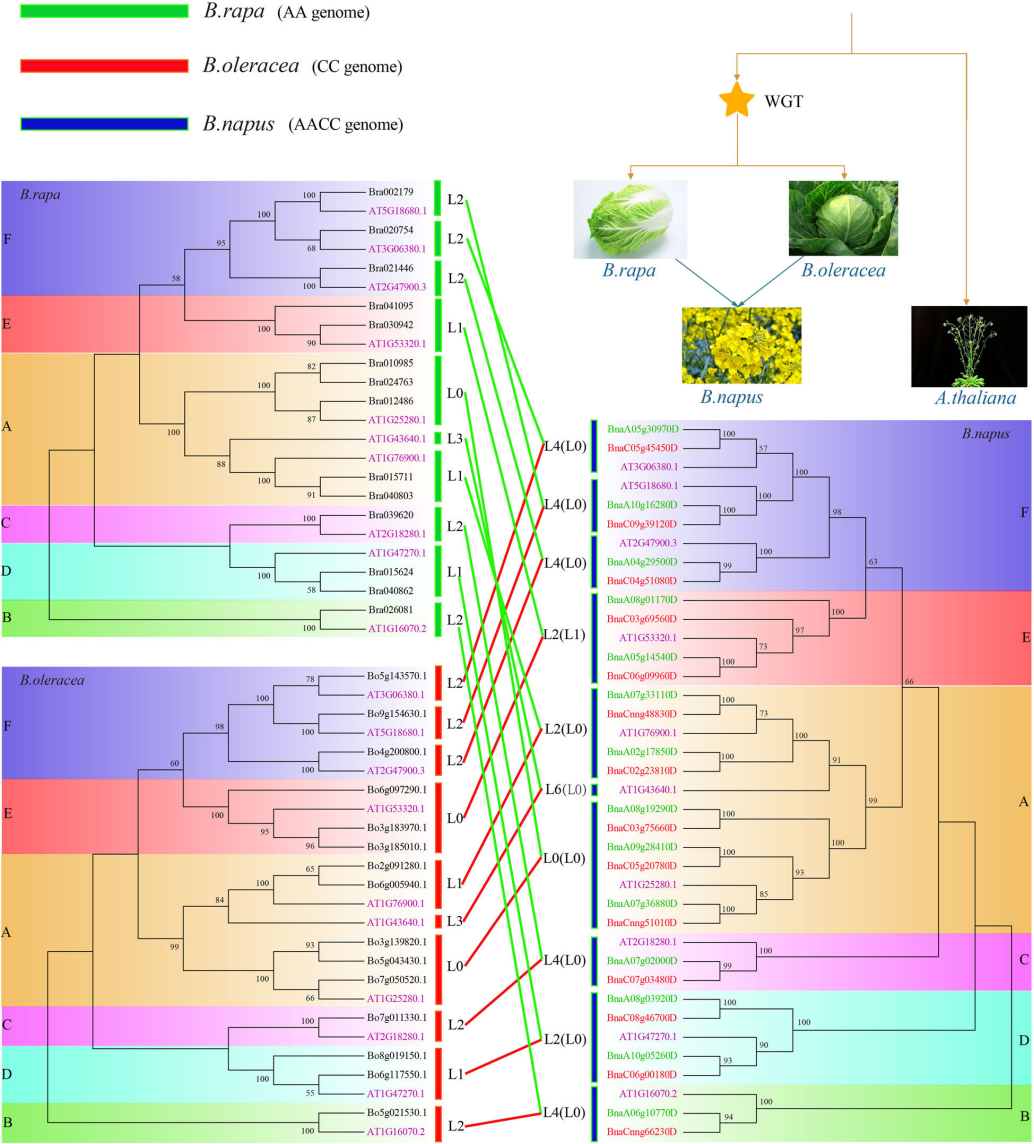
The duplication or loss analyses of *TLP* genes in *B. napus, B. rapa, B. oleracea* compared with *A. thaliana.* The “L”and “D” indicates the loss and duplication, respectively. The number after “L” and “D” represents the number of genes

We obtained quantitative changes in the number of *TLP* genes in different evolutionary stages based on the phylogenetic reconstruction (Fig. 4). In phylogenetic tree of *A. thaliana* and *B. rapa*, one *A. thaliana* gene should theoretically correspond to three genes of *B. rapa*, but we clearly saw that one *A. thaliana* gene corresponded to only one gene in *B. rapa* for GroupB, GroupC and GroupF, and two genes were lost. The gene (*AT1G25280*) in GroupA was completely retained after WGT in *B. rapa* (*AT1G25280* vs *Bra010985*, *Bra024763* and *Bra012486*), indicating that these genes might play a very important role in *B. rapa*. In particular, it might be a gene dosage effect, explaining the significant differences between *B. rapa* and *A. thaliana* for some certain traits. The gene corresponding to *AT1G43640* in GroupA was completed lost in *B. rapa*, indicating that this gene might not function in *B. rapa*. In GroupD and E, one gene was lost in *B. rapa* corresponding to *A. thaliana*.

In phylogenetic tree of *A. thaliana* and *B. oleracea* (Fig. 4), the loss of gene in GroupA, B, C, D, and F was consistent with that of *B. rapa*. In GroupE, three genes of *B. oleracea* were not lost (*AT1G53320* vs *Bo6g097290*, *Bo3g183970* and *Bo3g185010*). In *B. rapa*, there were only two copies of this gene in *A. thaliana*, and a gene loss occurs in GroupE.

In phylogenetic tree of *A. thaliana* and *B. napus* (Fig. 4), one *A. thaliana* gene corresponded to six *B. napus* genes. The *TLP* gene in *B. napus* had a lot of loss after WGT event. In fact, the loss number of each group varied from 2 to 6 genes. For example, the gene corresponding to *AT1G43640* had all been lost in *B. napus*. However, the six genes corresponding to *AT1G25280* were all retained in *B. napus.* In fact, based on the analysis of *B. oleracea* and *B. rapa*, it was clear that the loss of *TLP* gene did not occur directly in *B. napus*. The loss of *TLP* genes occurred during the WGD event of the diploid parents *B. oleracea* and *B. rapa*. The phylogenetic tree connection showed that the total number of genes in each group of *B. napus* has been evolved to be sum of the number of corresponding groups of *B. oleracea* and *B. rapa* (28 vs 14 + 15). Only in GroupE, the number of *B. rapa* relative to *A. thaliana* genes was lost (Ath: 1 vs Bra: 2), and *B. oleracea* gene was not lost (Ath: 1 vs Bol: 3). Therefore, there should be 5 *TLP* genes in GroupE of *B. napus.* However, we found that there were only 4 *TLP* genes in this group, which meant that 1 gene was lost after the formation of *B. napus*. Of course, there was also a case that we originally filtered out *BnaC09g39130D* from subgenome C, which was most likely from this group. However, a significant domain was loss in this gene, resulting in the failure to this group. In summary, we found that the genes in *B. napus* did not directly expand compared to their diploid parents *B. oleracea* and *B. rapa*. Thus, the expansion of this gene family of *B. napus* is an indirect expansion, that is, the expansion occurred in its two diploid parents.

### Gene expression pattern analysis of *TLP* gene family in *B. napus*

**Fig. 5:**
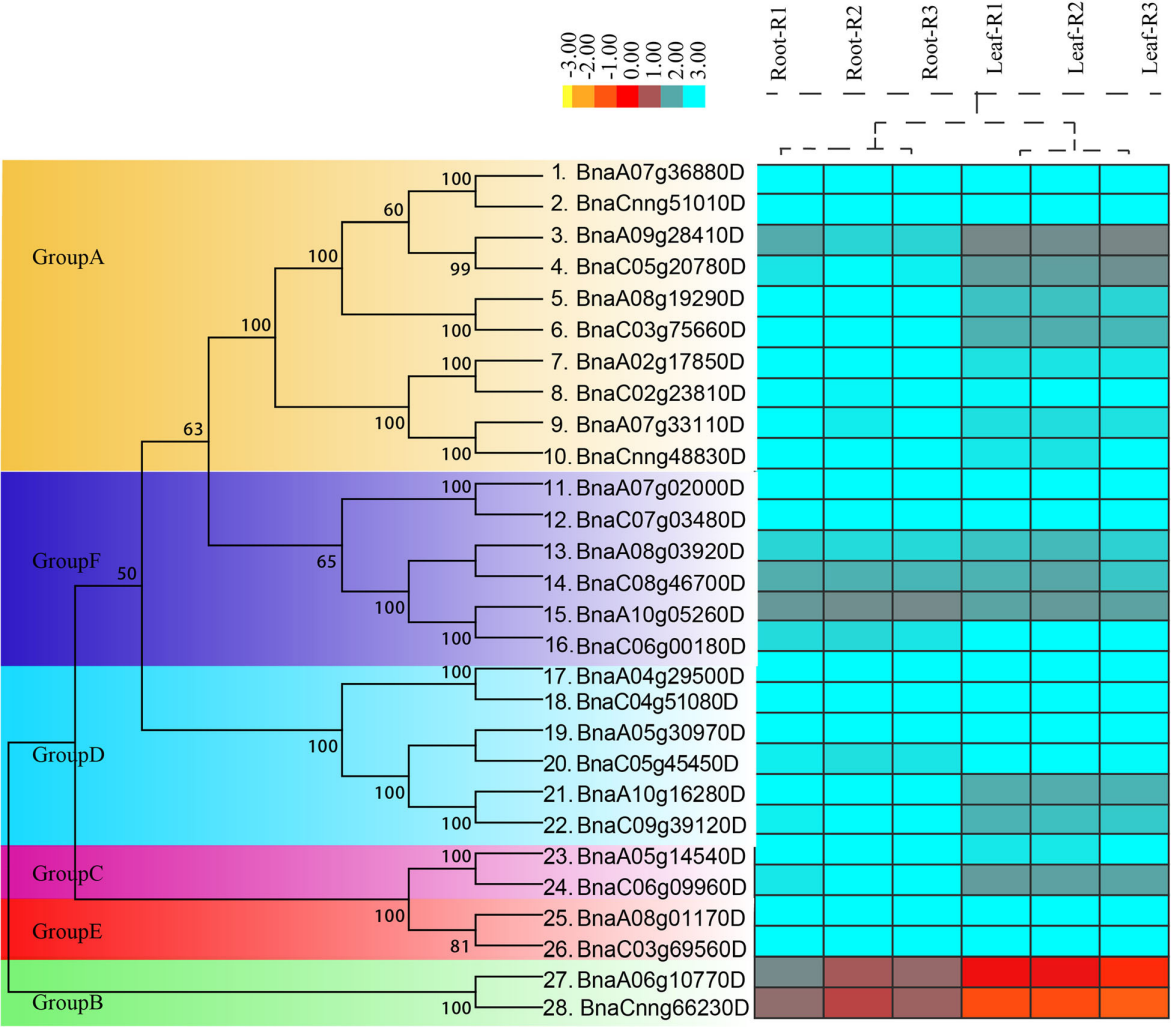
The expression hierarchical clustering of *TLP* genes in *B. napus*. The gene expression in roots (R1, R2, and R3 for three replicates) and leaves (L1, L2, and L3) were determined by RNA-seq. The expression values were calculated by RPKM (Reads Per Kilobase per Million mapped reads), and the expression values were log2 transformed

To explore the potential function of *TLP* family genes in different tissues of *B. napus*, the transcriptome data was used to calculate the expression of *TLP* family genes in two tissues, including roots and leaves. The expression levels were estimated by RPKM, and the deeper of the blue, the higher of the expression (Fig. 5, Table S5). The results showed that most of *TLP* genes had higher expression levels in roots and leaves except for the low expression levels of the two genes in GroupB. Of course, the expression patterns of some *TLP* genes in two tissues were slightly different. For example, the expression levels of *BnaA09g28410*, *BnaC05g20780D*, *BnaA10g16280D*, *BnaC09g39120D* and *BnaC06g09960D* in roots were higher than those in leaves.

**Fig. 6:**
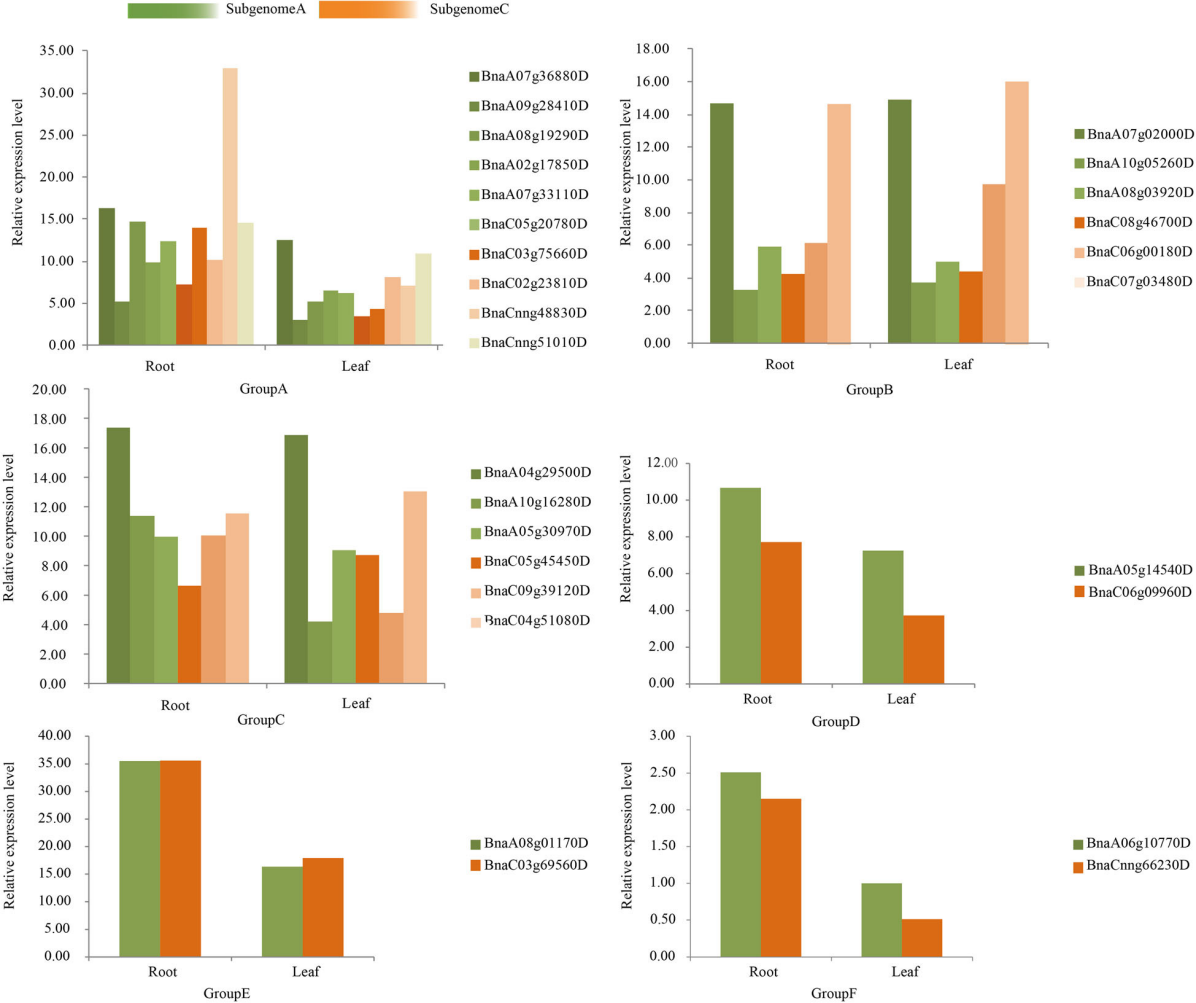
Histogram of *TLP* gene expression in root and leaf of in *B. napus*. Group A to Group F represent 6 groups classified by *TLP* genes. The genes located in subgenome A of *B. napus* were marked green, and the genes located subgenome C were marked red

In addition, we also compared the expression differences of *TLP* genes in roots and leaves in subgenome A and subgenome C for each group (Fig. 6). The results showed that the expression patterns of *TLP* genes between subgenome A and subgenome C were similar. *BnaCnng48830D* in GroupA was highly expressed compared to other genes. The expression of *BnaA09g28410D* and *BnaC05g20780D*, *BnaA08g19290D*, *BnaC03g75660D*, *BnaA10g16280D*, *BnaC06g09960D*, *BnaC09g39120D* in roots were significantly higher than that in leaves, indicating that these genes might play an important role in the morphogenesis of roots. The expression of *BnaA06g10770D* and *BnaCnng66230D* were extremely low in roots and leaves of *B. napus*. Several genes were also highly expressed in roots and leaves, such as *BnaA07g36880D* and *BnaCnng51010D*, *BnaA08g01170D* and *BnaC03g69560D*. These genes might be involved in the transcriptional regulation of various physiological and biochemical change in the whole growth and development cycle of *B. napus*. In conclusion, it was found that not only the group had similar conservative motifs, but also had similar expression patterns, which made the gene structure and function uniform.

## Discussion

### Systematical and comprehensive analyses of *TLP* gene family in *B. napus*

This study systematically compared and analyzed the *TLP* gene family of *B. napus* on the basis of predecessors. Up to now, we have systematically analyzed multiple gene families of *B. rapa* and *B. napus,* such as *BES1, AP2/ERF, CO-Like, bHLH, BES1, HSF*, *GARS*, and cold-related genes [36–46]. The methods, techniques, and experiences of these studies laid the foundation for an in-depth analysis of *TLP* gene family. In order to further analyze the evolutionary relationship between *B. napus* and other species homologous genes, this study constructed a phylogenetic tree of *B. napus* and *B. rapa*, *A. thaliana* and *B. oleracea TLP* family genes. In the structural analysis of *TLP* genes, it was found that the gene family structure of *B. napus* was relatively complicated. In the paralogous gene analysis, it was found that there were 63 pairs of paralogous genes in *B. napus*. So many paralogous genes also gave us a new understanding of the *TLP* gene family in *B. napus*.

### Evolution and expansion of *TLP* gene family in *Brassica* species

In the analysis of the duplication and loss of *TLP* gene family during evolution, we found a very interesting gene, the *A. thaliana AT1G25280* gene from GroupA group. The gene corresponded to 3, 3 and 6 *TLP* genes in *B. oleracea*, *B. rapa* and *B. napus*, respectively. It indicated that all copies of this gene are preserved after WGT in *Brassica* crops, which was in accordance with 1:3:6 duplication ratios. It indicated that they might have very important functions for the growth and development of *Brassica* and even in *B. napus*. Of course, the opposite evolutionary pattern was the *AT1G43640* gene of *A. thaliana*. Its homologous genes were not detected in *B. oleracea*, *B. rapa* and *B. napus*, that was, the gene was completely lost after WGT event. This indicated that the gene might not play any functional role for *Brassica* genus. In addition, this study found that the loss of *TLP* family genes was not directly in the *B. napus* genome through comparative analysis. It occured in the WGD process of the diploid parents *B. rapa* and *B. oleracea*. Therefore, the *TLP* family genes in *B. napus* did not directly expand compared to their two diploid parents. Thus, the expansion of this gene family in *B. napus* was an indirect expansion, that was, the expansion of this family genes in its two diploid parents.

In addition, we also identified *TLP* gene family in other *Brassica* species for comparative analyses. Totally, 42, 28, 14, 13, and 14 *TLP* genes were detected in the *B. napus* ‘ZS11’, *B. napus* ‘Tapidor’, *B. rapa* ‘Z1’ (yellow sarson), *B. oleracea* ‘kale-like’, and *B. oleracea* ‘HDEM’ (broccoli) (Figs. S1, S2, S3, S4 and S5). We found that the number of the *TLP* genes in these species except *B. napus* ‘ZS11’ was similar with the 3 *Brassica* species used in our study. The *TLP* genes in *B. napus* ‘ZS11’ was more than that in other *B. napus* species*.* This might be due to genome assembly and gene prediction, because the number of genome-wide genes in *B. napus* ‘ZS11’ (123,465) was also more than that in *B. napus* ‘Tapidor’ (70,162) and *B. napus* ‘Darmor-bzh’ (101,040).

Furthermore, we have performed the analysis of gene duplication and loss. It was found that the evolution pattern of most *TLP* genes in these species had similar patterns of duplication and loss as the three *Brassica* species we studied (Figs. S1, S2, S3, S4 and S5). However, there were some inconsistencies among these species. For example, compared with the *AT1G25280.1* gene in Arabidopsis, no homologous gene was lost in *B. napus* ‘Darmor-bzh’ and *B. napus* ‘ZS11’, while one gene was lost in *B. napus* ‘Tapidor’. Compared with the *AT1G47270.1*, two gene were lost in *B. napus* ‘Darmor-bzh’ and *B. napus* ‘Tapidor ‘, while three genes were lost in *B. napus* ‘ZS11’. Compared to the *AT1G16070.2*, four genes were lost in *B. napus* ‘Darmor-bzh’ and *B. napus* ‘ZS11’, while only one gene was lost in *B. napus* ‘Tapidor ‘.

### Exploring *TLP* gene function in more species

In recent years, with the deepening of *TLP* gene research, it has been found that they played a major role in plant growth, development and stress response. Studying the distribution, gene structure and expression analysis of *TLP* family genes in plant was great significant for further study of their function. In previous studies on *TLP* genes, 11 *TLP* family members have been found in *A. thaliana*, 14 family members in *O. sativa*, and 15 *TLP* family members in maize. The widespread presence of *TLP* genes indicated that they played an extremely important role in the life process [28, 29]. For example, a partial disease phenotype was produced when a genetic mutation occurred in a *TLP* gene. Although the function of *TLP* family genes in animals and plants has not yet been fully clarified, some research results have been obtained on the mining and research of their function and structure. The highly conserved nature of *TLP* domain indicated that they had important physiological functions in multicellular eukaryotes [16–18]. In particular, studies of plant *TLP* gene family have revealed that multiple *TLP* genes were involved in plant responses to biotic and abiotic stresses [15, 27]. This indicated that *TLP* genes could be used as candidate genes for plant stress-resistant breeding and applied to plant resistance breeding. The comparative genomics study of *TLP* gene family of *B. napus* in this research system will inevitably lay a solid foundation for the functional study of *TLP* gene family.

## Conclusions

In conclusion, we comprehensively analyzed the evolutionary pattern, gene structure, orthologous and paralogous genes, duplication type, gene synteny, gene duplication or losses, and gene expression pattern of *TLP* genes in *B. napus* and other Brassicaceae species. A total of 68 *TLP* genes were identified in these species, and 28 genes were identified in *B. napus.* Identification of these transcription factor genes was likely to assist in clarifying the molecular genetics basis for *B. napus* genetic improvement, and also provided the functional gene resources for transgenic research. Until now, few genes representing this gene family have been characterized in detail from *B. napus.* Therefore, this is the first comprehensive and systematic analyses of *TLP* gene family in *B. napus.* This study provides useful resources for future studies on the structure and function of *TLP* genes in *B. napus.* In addition, our analyses showed that the directly expansion of *TLP* genes existed in *B. napus,* and the real *TLP* expansion occurred in its diploid parents *B. rapa* and *B. oleracea*. This study may also facilitate our understanding of the effect of duplication or losses during the evolution of *B. napus* or others polyploidy.

## Methods

### Collection of genomic data and identification of *TLP* gene family

The *A. thaliana* genome-related data used in this study was derived from Tair website (Tair10, https://www.arabidopsis.org). *B. napus* ‘Darmor-bzh’ (v5.0) and *B. rapa* ‘Chiifu’ (v3.0) genomic data were derived from BRAD database (http://brassicadb.org/brad/index.php) [1]. *B. oleracea* var. capitata line 02–12 genome (v1.1) datasets were derived from Bolbase database (http://www.ocri-genomics.org/bolbase/index.html) [4]. The protein sequences of *B. rapa* ‘Z1’ (yellow sarson) and *B. oleracea* ‘HDEM’ (broccoli) were downloaded from genoscope (http://www.genoscope.cns.fr/externe/plants/chromosomes.html) [47]. The genome sequences of *B. oleracea* kale-like type TO1000 were downloaded from EMBL [6]. The genome sequences of *B. napus* ‘ZS11’ (v2.0) were derived from NCBI (https://www.ncbi.nlm.nih.gov/genome/203), and *B. napus* ‘Tapidor’ (v6.3) genome sequences were downloaded from applied bioinformatics group (http://appliedbioinformatics.com.au/index.php/Darmor_Tapidor) [48]. The Pfam (http://pfam.sanger.ac.uk) database was used to perform domain search on the amino acid sequences of the downloaded species, and the genes containing “*TLP*” domain were extracted by the self-programmed Perl program (PF01167). At the same time, in order to ensure the accuracy of the results, the SMART (http://smart.embl-heidelberg.de/smart/change_mode.pl) and CDD (https://www.ncbi.nlm.nih.gov/Structure/cdd) databases were further used to perform domain validation on the genes identified above [30, 49].

### Gene structural and conservative motif analyses of *TLP* genes in *B. napus*

Information on the location of *TLP* genes in *B. napus*, such as chromosome, genomic location, CDS, protein sequence, etc., were obtained from the databases mentioned above. The gene structure was analyzed by the online tool GSDS (http://gss.cbi.pku.edu.cn/index.php) [50]. It could show the position of introns, exons, and un-translated regions (UTRs) of the gene. The gff file of *TLP* family genes was submitted to the GSDS program to obtain a schematic diagram of the gene structure. The online analysis software MEME (http://meme.nbcr.net/meme4-1/cgi-bin/meme.cgi) was used to analyze the amino acid sequence of *TLP* genes in *B. napus*, and 6 motifs were obtained and used for further analyses.

### Evolution analysis of *TLP* gene family in *B. napus*

The ClustalW program was used to perform multiple alignments of the amino acid sequences of the *TLP* gene family using default parameter values (https://www.genome.jp/tools-bin/clustalw). The incomplete reading frame sequences and redundant sequences were manually removed. The phylogenetic tree of *TLP* gene family was constructed with Neighbor-Joining (NJ) method using Mega7.0 software (http://megasoftware.net) [51, 52]. The evolutionary tree was evaluated by Bootstrap method, and the value was set as1000 [51]. The position information on the chromosome of *TLP* family genes was extracted from gff file, and the chromosome map was drawn using Perl program.

### Identification of orthologous and paralogous genes

The orthologous and paralogous relationships between the *TLP* genes of *B. napus* and *A. thaliana*, *B. rapa, B. oleracea* were identified using OrthoMCL software (http://orthomcl.org/orthomcl/) [53]. Images of the relationships between the paralogous and orthologous of *A. thaliana, B. rapa, B. oleracea*, and *B. napus* were drawn using Circos software (http://circos.ca/) [54].

### Ka/Ks calculation and dating the divergent time

To estimate the divergence of orthologous genes, the sequences of orthologous *TLP* gene pairs between *B. napus* and other three species (*A. thaliana*, *B. rapa*, *B. oleracea*) were aligned using ClustalW (https://www.genome.jp/tools-bin/clustalw). Then, the nonsynonymous rate (Ka), synonymous rate (Ks), and evolutionary constraint (Ka/Ks) between the orthologous gene pairs were calculated according to their coding sequence alignments by using Nei-Gojobori method implemented in the Ka/Ks_calculator program [55, 56]. The orthologous gene pairs with Ks < 1 were used for the divergence time estimation based on the neutral substitution rate 1.5 × 10^− 8^ substitutions per site per year [57].

### Analysis of expression pattern of *TLP* gene family in *B. napus*

The RNA-seq data was used to analyze *TLP* gene expression patterns in leaves and roots in *B. napus* [58]. This dataset contained three replicates, and RPKM value was log10 transformed. The average value was used to compare the expression level of *TLP* genes between subgenome A and C, or root and leaf. The heatmap package (https://cran.r-project.org/web/packages/pheatmap/index.html) of R was used to draw the expression heatmap.

## Supplementary information

**Additional file 1: Table S1.** The list of orthologous TLP gene pairs between *B. napus*, *A. thaliana*, *B. rapa*, and *B. oleracea*. **Table S2.** The list of paralogous *TLP* gene pairs in each of other examined species. **Table S3.** Ka/Ks calculation and divergent time of the orthologous gene pairs between *B. napus* and other 3 species. **Table S4.** The duplicated type of *TLP* genes in *B. napus* and other three brassicaceae species. The 0 to 4 indicate the singleton, dispersed, proximal, tandem, WGD duplication type, respectively. **Table S5.** The expression level of the TLP genes in root and leaf for *B. napus*. The gene expression was determined by the RNA-Seq data (RPKM).

**Additional file 2: Figure S1.** The duplication or loss analyses of TLP genes in *B. napus* ‘ZS11’ compared with *A. thaliana.* The “L”and “D” indicates the loss and duplication, respectively. The number after “L” and “D” represents the number of genes. **Figure S2.** The duplication or loss analyses of TLP genes in *B. napus* ‘Tapidor’ compared with *A. thaliana.* The “L”and “D” indicates the loss and duplication, respectively. The number after “L” and “D” represents the number of genes. **Figure S3.** The duplication or loss analyses of TLP genes in *B. oleracea* ‘HDEM’ compared with *A. thaliana.* The “L”and “D” indicates the loss and duplication, respectively. The number after “L” and “D” represents the number of genes. **Figure S4.** The duplication or loss analyses of TLP genes in *B. oleracea* ‘kale-like’ compared with *A. thaliana.* The “L”and “D” indicates the loss and duplication, respectively. The number after “L” and “D” represents the number of genes. **Figure S5.** The duplication or loss analyses of TLP genes in *B. rapa* ‘Z1’ compared with *A. thaliana.* The “L”and “D” indicates the loss and duplication, respectively. The number after “L” and “D” represents the number of genes.

## Data Availability

All data generated or analysed during this study were included in this published article and the additional files. *A. thaliana* genome: https://www.arabidopsis.org; *B. napus* ‘Darmor-bzh’ and *B. rapa* ‘Chiifu’ genome: http://brassicadb.org/brad/index.php; *B. napus* ‘ZS11’ genome: https://www.ncbi.nlm.nih.gov/genome/203; *B. napus* ‘Tapidor’ genome: http://appliedbioinformatics.com.au/index.php/Darmor_Tapidor; *B. rapa* ‘Z1’ genome: http://www.genoscope.cns.fr/externe/plants/chromosomes.html; *B. oleracea* var. capitata line 02–12 genome: http://www.ocri-genomics.org/bolbase/index.html; *B. oleracea* ‘HDEM’: http://www.genoscope.cns.fr/externe/plants/chromosomes.html.

## Abbreviations

*TLP*: Tubby-like proteins
SKP1: S-phase kinase-associated protein 1
WGD: Whole-genome duplication
WGT: Whole-genome triplication
CDS: Coding domain sequence
