# Supplementary material for: Identification, evolution and expression analyses of whole genome-wide TLP gene family in Brassica napus
Source: BMC Genomics. 2020 Mar 30;21:264. doi: 10.1186/s12864-020-6678-x (PMC7106719; doi:10.1186/s12864-020-6678-x)
Supplement: Supplementary file 2 — Additional file 2: Figure S1. The duplication or loss analyses of TLP genes in B. napus ‘ZS11’ compared with A. thaliana. The “L”and “D” indicates the loss and duplication, respectively. The number after “L” and “D” represents the number of genes. Figure S2. The duplication or loss analyses of TLP genes in B. napus ‘Tapidor’ compared with A. thaliana. The “L”and “D” indicates the loss and duplication, respectively. The number after “L” and “D” represents the number of genes. Figure S3. The duplication or loss analyses of TLP genes in B. oleracea ‘HDEM’ compared with A. thaliana. The “L”and “D” indicates the loss and duplication, respectively. The number after “L” and “D” represents the number of genes. Figure S4. The duplication or loss analyses of TLP genes in B. oleracea ‘kale-like’ compared with A. thaliana. The “L”and “D” indicates the loss and duplication, respectively. The number after “L” and “D” represents the number of genes. Figure S5. The duplication or loss analyses of TLP genes in B. rapa ‘Z1’ compared with A. thaliana. The “L”and “D” indicates the loss and duplication, respectively. The number after “L” and “D” represents the number of genes. [file 12864_2020_6678_MOESM2_ESM.docx]

**Supplementary Figure 1-5**

**
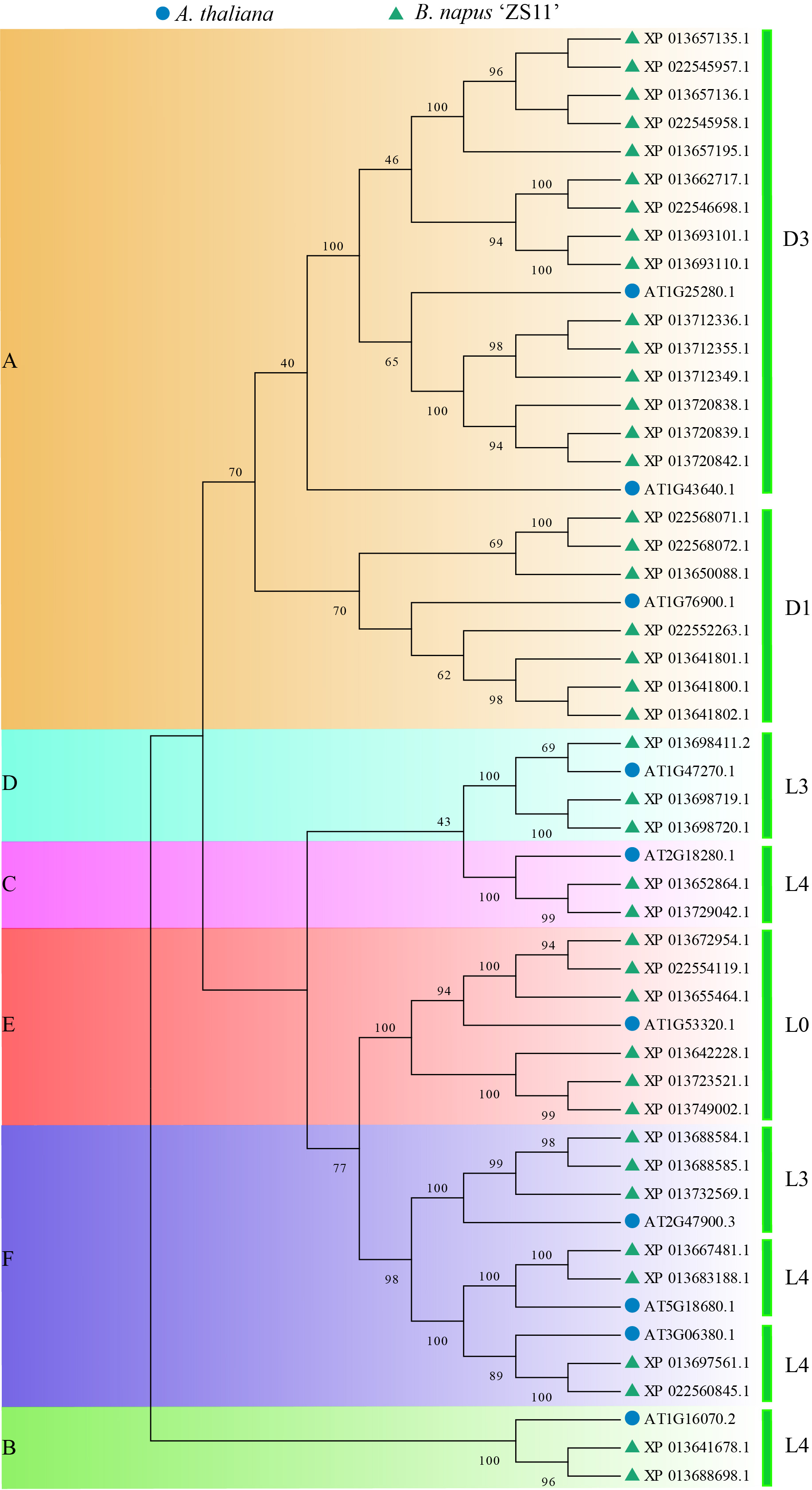
**

**Supplementary Figure 1.** The duplication or loss analyses of TLP genes in *B. napus* ‘ZS11’ compared with *A. thaliana.* The “L”and “D” indicates the loss and duplication, respectively. The number after “L” and “D” represents the number of genes.

**
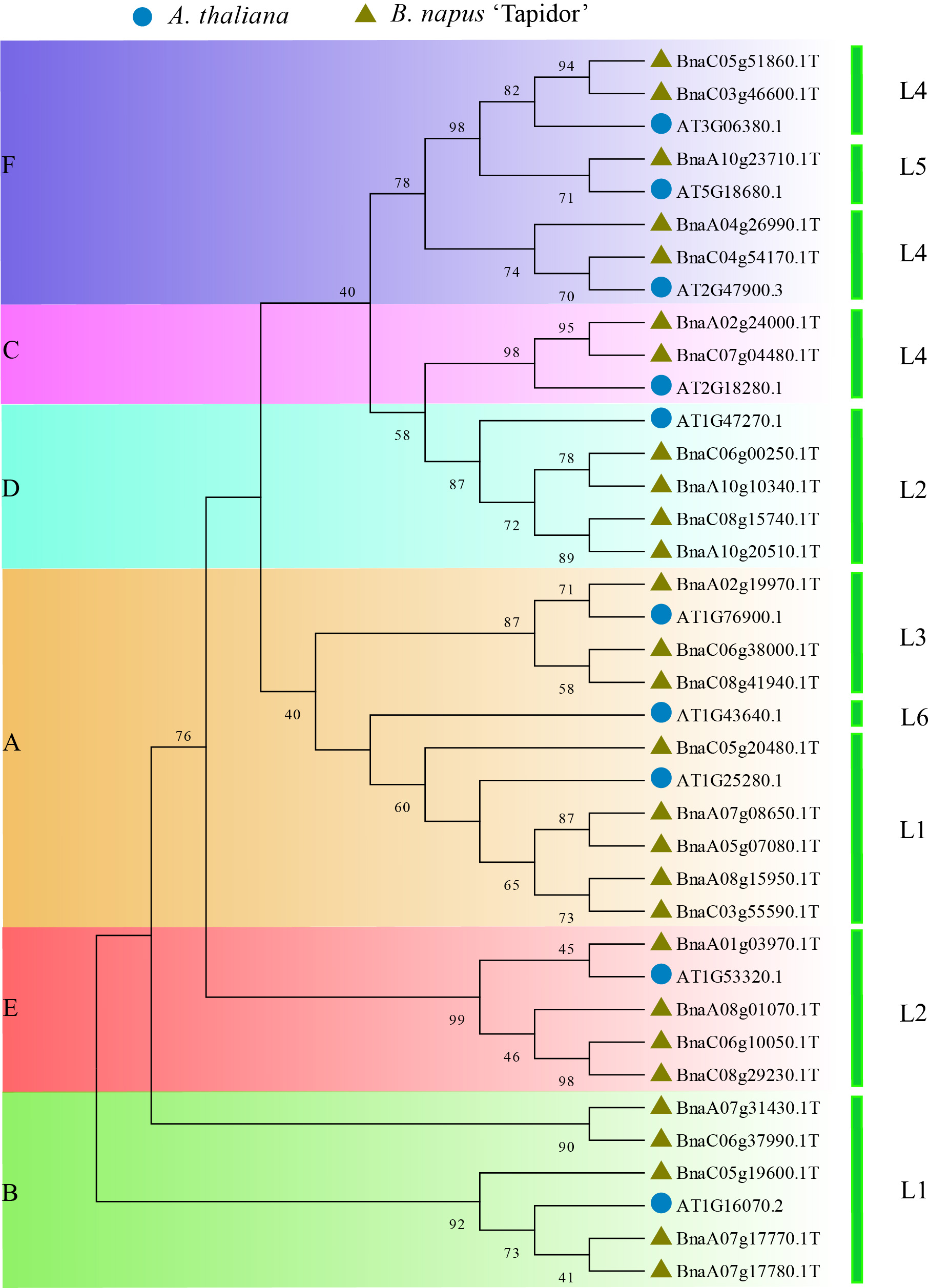
**

**Supplementary Figure 2.** The duplication or loss analyses of TLP genes in *B. napus* ‘Tapidor’

compared with *A. thaliana.* The “L”and “D” indicates the loss and duplication, respectively. The number after “L” and “D” represents the number of genes.


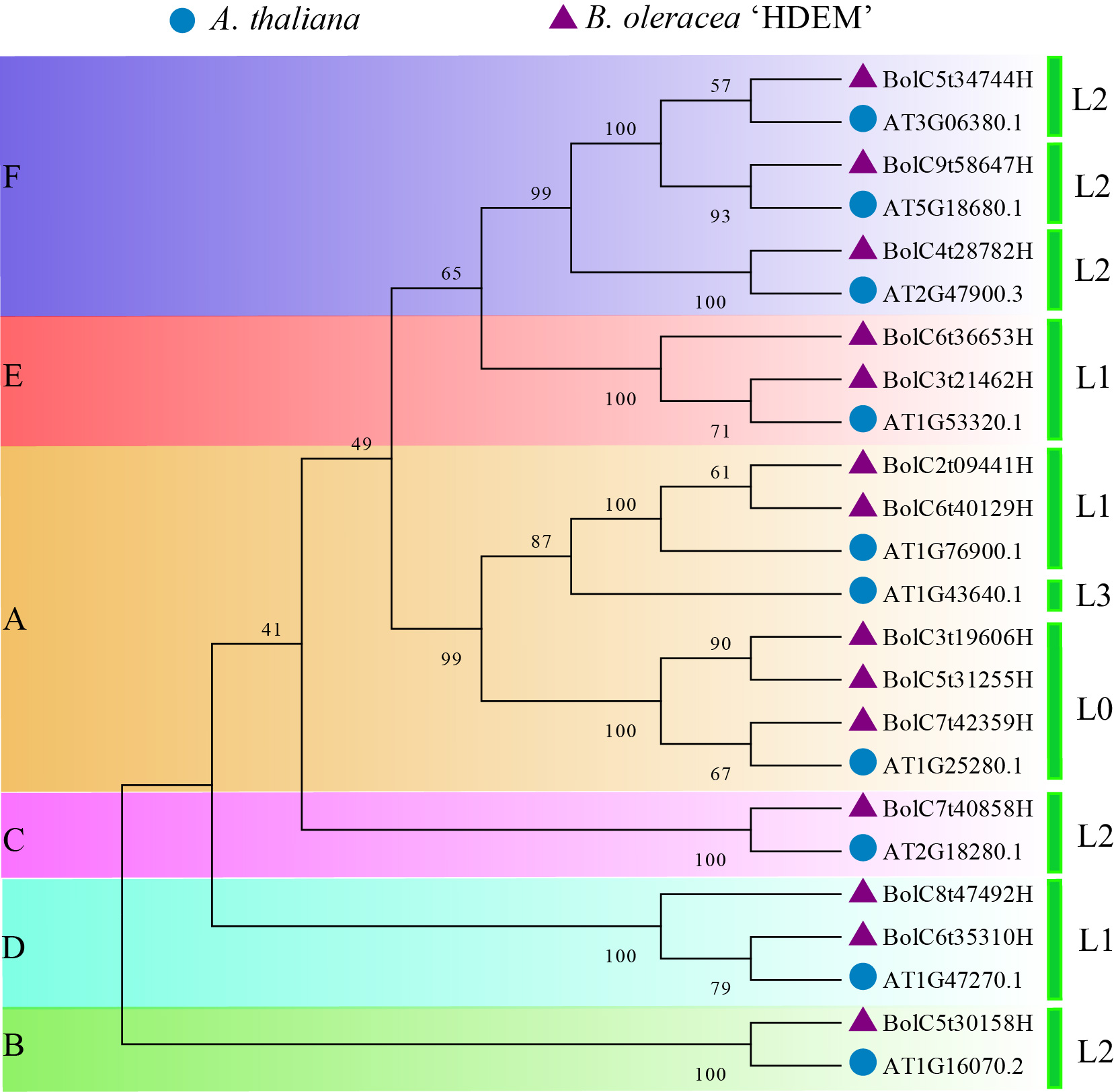


**Supplementary Figure 3.** The duplication or loss analyses of TLP genes in *B. oleracea* ‘HDEM’

compared with *A. thaliana.* The “L”and “D” indicates the loss and duplication, respectively. The number after “L” and “D” represents the number of genes.


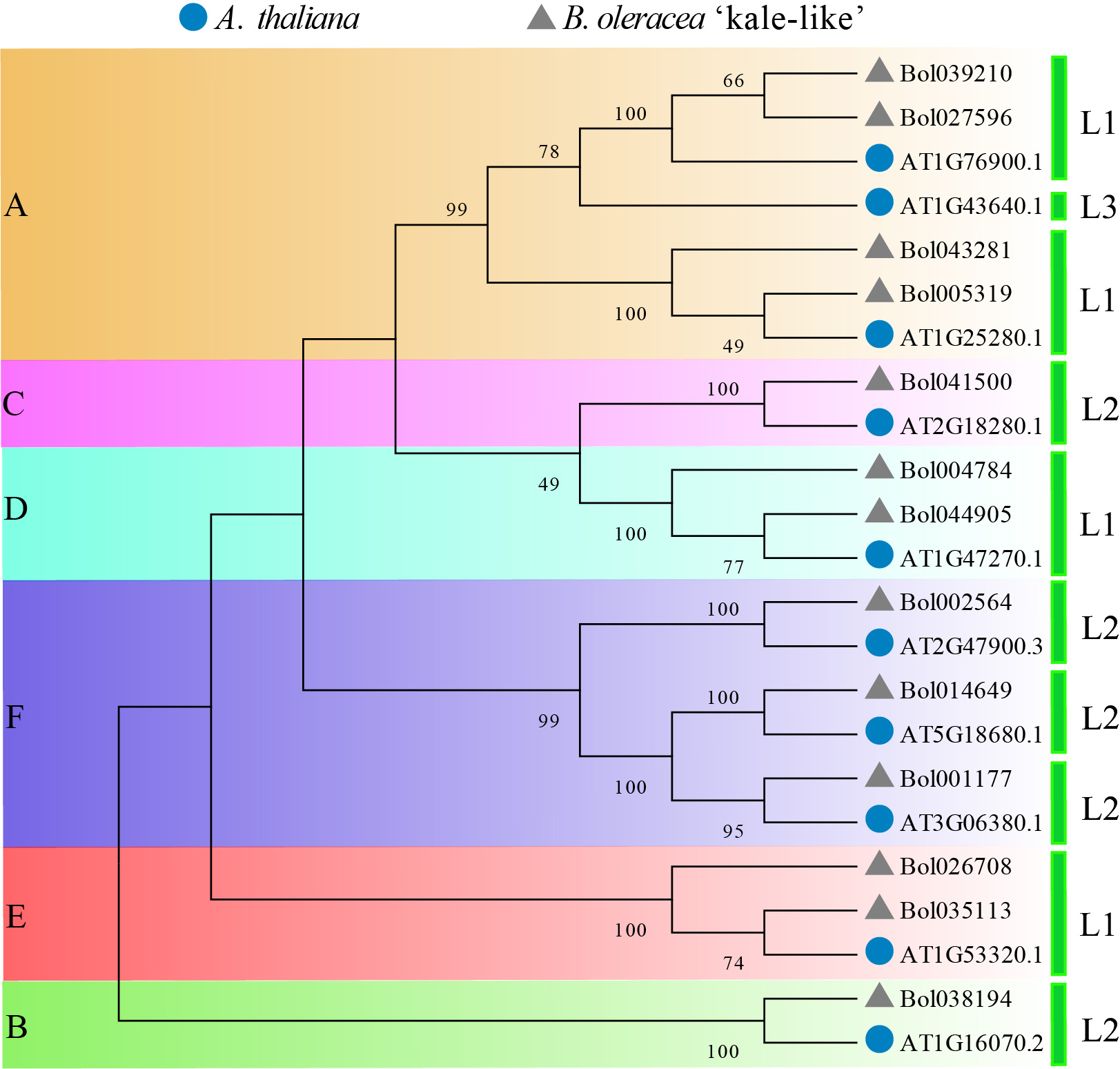


**Supplementary Figure 4.** The duplication or loss analyses of TLP genes in *B. oleracea* ‘kale-like’

compared with *A. thaliana.* The “L”and “D” indicates the loss and duplication, respectively. The number after “L” and “D” represents the number of genes.


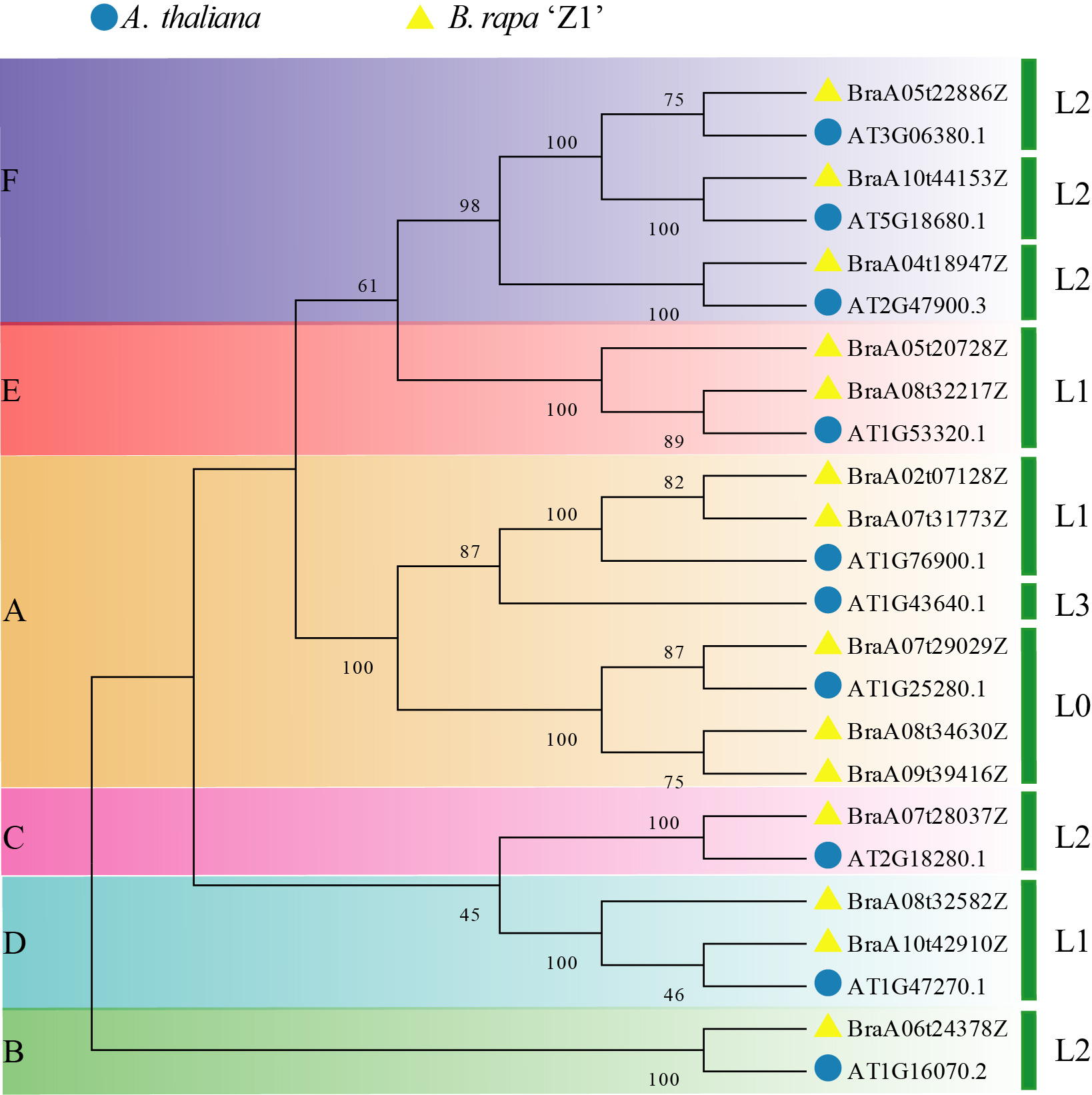


**Supplementary Figure 5.** The duplication or loss analyses of TLP genes in *B. rapa* ‘Z1’

compared with *A. thaliana.* The “L”and “D” indicates the loss and duplication, respectively. The number after “L” and “D” represents the number of genes.
